# Visualization of bone details in a novel photon-counting dual-source CT scanner—comparison with energy-integrating CT

**DOI:** 10.1007/s00330-021-08441-4

**Published:** 2021-12-22

**Authors:** Stefanie J. Bette, Franziska M. Braun, Mark Haerting, Josua A. Decker, Jan H. Luitjens, Christian Scheurig-Muenkler, Thomas J. Kroencke, Florian Schwarz

**Affiliations:** Clinic for Diagnostic and Interventional Radiology and Neuroradiology, University Hospital Augsburg, Stenglinstr. 2, 86156 Augsburg, Germany

**Keywords:** Multidetector computed tomography, X-ray computed tomography, Bone and bones

## Abstract

**Objectives:**

Photon-counting detector CT (PCD-CT) promises a leap in spatial resolution due to smaller detector pixel sizes than implemented in energy-integrating detector CTs (EID-CT). Our objective was to compare the visualization of smallest bone details between PCD-CT and EID-CT using a mouse as a specimen.

**Materials and methods:**

Two euthanized mice were scanned at a 20-slice EID-CT and a dual-source PCD-CT in single-pixel mode at various CTDI_Vol_ values. Image noise and signal-to-noise ratio (SNR) were evaluated using repeated ROI measurements. Edge sharpness of bones was compared by the maximal slope within CT value plots along sampling lines intersecting predefined bones of the spine. Two readers evaluated bone detail visualization at four regions of the spine on a three-point Likert scale at various CTDI_Vol_’s. Two radiologists selected the series with better detail visualization among each of 20 SNR-matched pairs of EID-CT and PCD-CT series.

**Results:**

In CTDI_Vol_-matched scans, PCD-CT series showed significantly lower image noise (Noise_*CTDI*=*5 mGy*_: 16.27 ± 1.39 vs. 23.46 ± 0.96 HU, *p* < 0.01), higher SNR (SNR_*CTDI*=*5 mGy*_: 20.57 ± 1.89 vs. 14.00 ± 0.66, *p* < 0.01), and higher edge sharpness (Edge Slope_lumbar spine_: 981 ± 160 vs. 608 ± 146 HU/mm, *p* < 0.01) than EID-CT series. Two radiologists considered the delineation of bone details as feasible at consistently lower CTDI_Vol_ values at PCD-CT than at EID-CT. In comparison of SNR-matched reconstructions, PCD-CT series were still considered superior in almost all cases.

**Conclusions:**

In this head-to-head comparison, PCD-CT showed superior objective and subjective image quality characteristics over EID-CT for the delineation of tiniest bone details. Even in SNR-matched pairs (acquired at different CTDI_Vol_’s), PCD-CT was strongly preferred by radiologists.

**Key Points:**

• *In dose-matched scans, photon-counting detector CT series showed significantly less image noise, higher signal-to-noise ratio, and higher edge sharpness than energy-integrating detector CT series*.

• *Human observers considered the delineation of tiny bone details as feasible at much lower dose levels in photon-counting detector CT than in energy-integrating detector CT*.

• *In direct comparison of series matched for signal-to-noise ratio, photon-counting detector CT series were considered superior in almost all cases*.

**Supplementary Information:**

The online version contains supplementary material available at 10.1007/s00330-021-08441-4.

## Introduction

Computed tomography (CT) is the most widely used tomographic imaging modality worldwide due to its short acquisition times, high technical reliability, and ubiquitous availability. CT scanners in clinical use today are equipped with energy-integrating detectors (EID) which are based on an indirect detection of X-ray photons after conversion into light in scintillator ceramics.

Very recently, photon-counting detectors (PCDs) have become available for clinical CT. Detecting charge separation in semiconductors induced by X-ray photons, PCDs directly convert X-rays into an electric signal. This confers substantial theoretical benefits such as improved spatial resolution due to smaller detector pixel size, elimination of electronic noise, and intrinsic spectral sensitivity [1, 2].

For maximal spatial resolution, a single-pixel acquisition mode is routinely available on the latest PCD-CT models. This mode discerns individual detector pixels and should considerably improve visualization of finest anatomic details such as in bone or in the lungs beyond that reported for earlier PCD-CT prototypes which aggregated two pixels for ultra-high-resolution (UHR) acquisitions [3, 4]. Previous studies on earlier PCD-CT prototypes have compared PCD-CT in UHR mode with EID-CT in phantoms and cadaveric specimen such as wrists and observed higher signal-to-noise ratio (SNR) of PCD-CT in computed tomography dose index (CTDI_Vol_)-equivalent acquisitions hinting at considerable dose-saving potential of the new technology [5, 6]. Uncertainty remains, however, to what extent this SNR advantage translates into improved visualization of anatomic details and whether there is a dose-dependency of this effect.

This study reports our comprehensive analyses of bone detail visualization on a novel dual-source PCD-CT in comparison with a modern EID-CT at various CTDI_Vol_ values using a mouse as a specimen. Because of the very small size of its skeleton, highly standardizable skeletal reference regions of different inherent complexity could be defined making the mouse an ideal model for our analysis. We systematically quantified image noise, signal-to-noise ratio, and edge sharpness of bones and compared visualization of bone details at different CTDI_Vol_’s between PCD-CT and EID-CT. Finally, observer preference in SNR-matched pairs of PCD-CT and EID-CT series was evaluated.

## Materials and methods

### Scanning of specimen

Two euthanized mice were purchased from a pet food store and positioned inside a sealed plastic box for CT scanning. By changing the position of the box and the table height, the mice were aligned in the isocenter and perpendicularly to the patient table.

The mice were sequentially scanned on two CT scanners (A: a 20-slice MDCT with energy-integrating detector (EID-CT): Somatom AS20, Siemens Healthineers, Erlangen, Germany; B: a novel photon-counting dual-source CT, NAEOTOM Alpha, Siemens Healthineers (PCD-CT)) using protocols optimized for bone imaging and identical computed tomography dose index settings (CTDI_vol(32 cm)_) at 120 kV. For EID-CT, collimation was 0.6 × 20 with a rotation time of 1.0 s and a spiral pitch of 0.8. For PCD-CT, collimation was 0.2 × 120 with a rotation time of 0.5 s and a spiral pitch of 0.2. For PCD-CT acquisitions, the routinely available single-pixel acquisition mode was selected. Scans were performed sequentially at different CTDI_Vol_’s ranging from 2.2 to 20 mGy. Tube modulation was disabled for all scans.

### Image reconstruction and postprocessing

For comparison between scanners, kernels with high spatial resolution and closest similarity in modulation transfer function were selected (B75h for EID-CT and Hr68 for PCD-CT, data provided by Siemens Healthineers). For PCD-CT, additional reconstructions using a Hr98 Kernel were generated for illustration purposes only. All reconstructions used filtered back projection with a quadratic field of view (11.4 cm) and a matrix of 512 × 512.

**Fig. 1:**
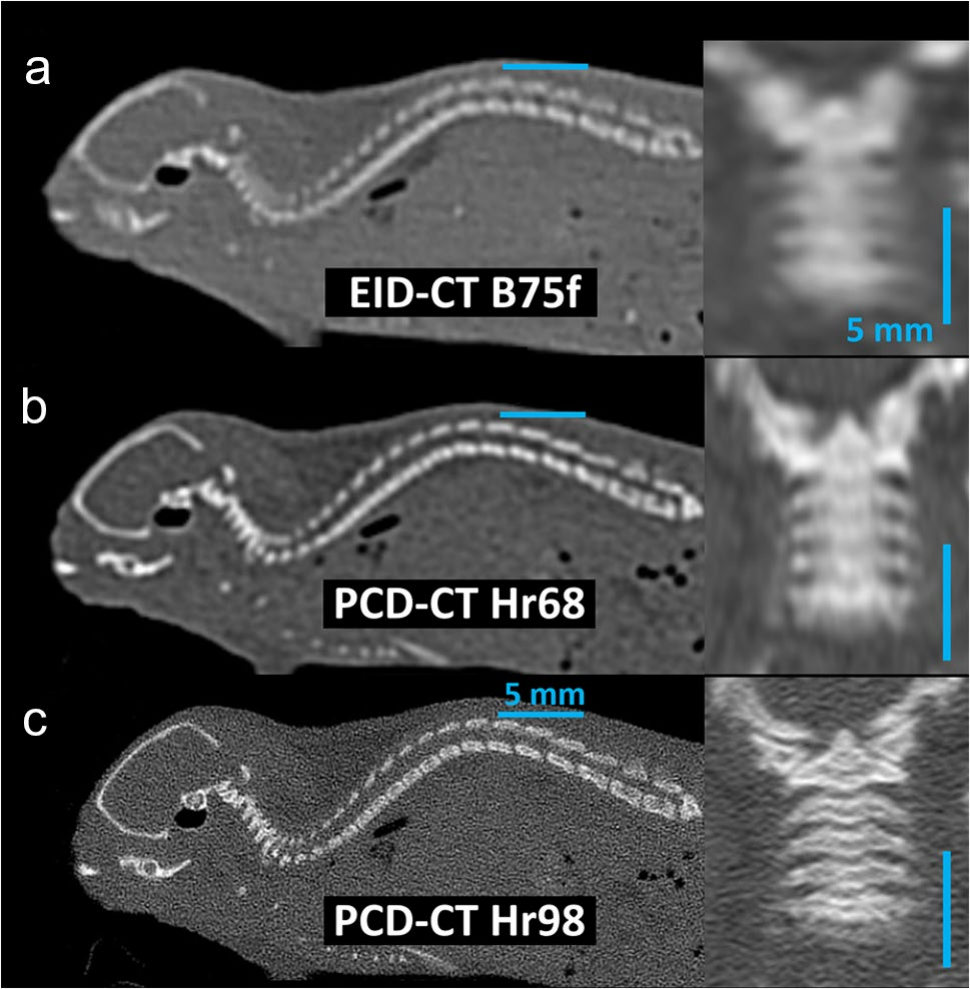
Reconstructed series used for analysis. Sagittal reconstructions of the whole spine (slice thickness: 0.2 mm) and coronal reformations of the cervical spine (slice thickness: 0.6 mm) at EID-CT B75f (**a**), PCD-CT Hr68 (**b**), and PCD-CT Hr98 (**c**) at CTDI_Vol_ = 15 mGy. CTDI_Vol_, volumetric CT dose index; EID-CT, energy-integrating detector CT; PCD-CT, photon-counting detector CT; B75h, Hr68, and Hr98 are abbreviations for specific reconstruction algorithms

For noise measurements, datasets were reconstructed with 1-mm slice thickness and 1-mm increment. For the evaluation of bone detail visualization, the minimal available slice thickness was chosen: 0.6 mm with an increment of 0.1 mm for EID-CT and 0.2 mm with an increment of 0.1 mm for PCD-CT, respectively. For better comparability, 0.6-mm EID-CT images were reformatted to a slice thickness of 0.2 mm with 0.1-mm increment using commercially available software (SyngoVia VB60, Siemens Healthineers). Furthermore, identical coronal reformations of the cervical spine were generated with a slice thickness of 0.6 mm and an increment of 0.1 mm (Fig. 1, Video 1).

### Quantification of image noise, signal-to-noise ratio, and edge sharpness

Image noise was measured using the open-source software *Fiji* [7]. Three identical regions of interest (ROIs) were defined in a large volume of air and copied to all 1.0-mm series. Standard deviation of CT values in air was used to approximate image noise and reported as mean of the measurements in the three ROIs.

SNR was quantified using the ratio between the CT values in bone and the standard deviation of CT values in air on 0.2-mm series: four 1.5 mm^2^ ROIs were defined on parasagittal planes in the lumbar vertebrae L2 and L3 and three large ROIs within air. Mean CT values of bone and mean SD of CT values in air were recorded.

Edge sharpness of bone surfaces was quantified using CT value plots along two sampling lines manually drawn at defined positions on the 0.2-mm reconstructions: in parasagittal planes along the cervical spine axis, intersecting the transverse processes; and in the median plane, perpendicularly to the thoracic or lumbar spinal canal intersecting the dorsal lamina. For the cervical spine, three measurements of edge sharpness of the transverse processes of C2 and C3 were performed for each CTDI_Vol_ value. For the thoracic and lumbar spine, three measurements were performed at each of six vertebrae (Th11–L4) for each CTDI_Vol_ value. As a measure for edge sharpness, the slope of the CT value plot at the bone surface was used, defined as the difference of CT values per millimeter:

$$\mathrm{Edge}\;\mathrm{Slope}=\frac{\left|\Delta\;\mathrm{CT}\;\mathrm{Values}\;\left(\mathrm{HU}\right)\right|}{\mathrm{Length}\;\left(\mathrm{mm}\right)}$$

### Evaluation of bone detail visualization

**Fig. 2:**
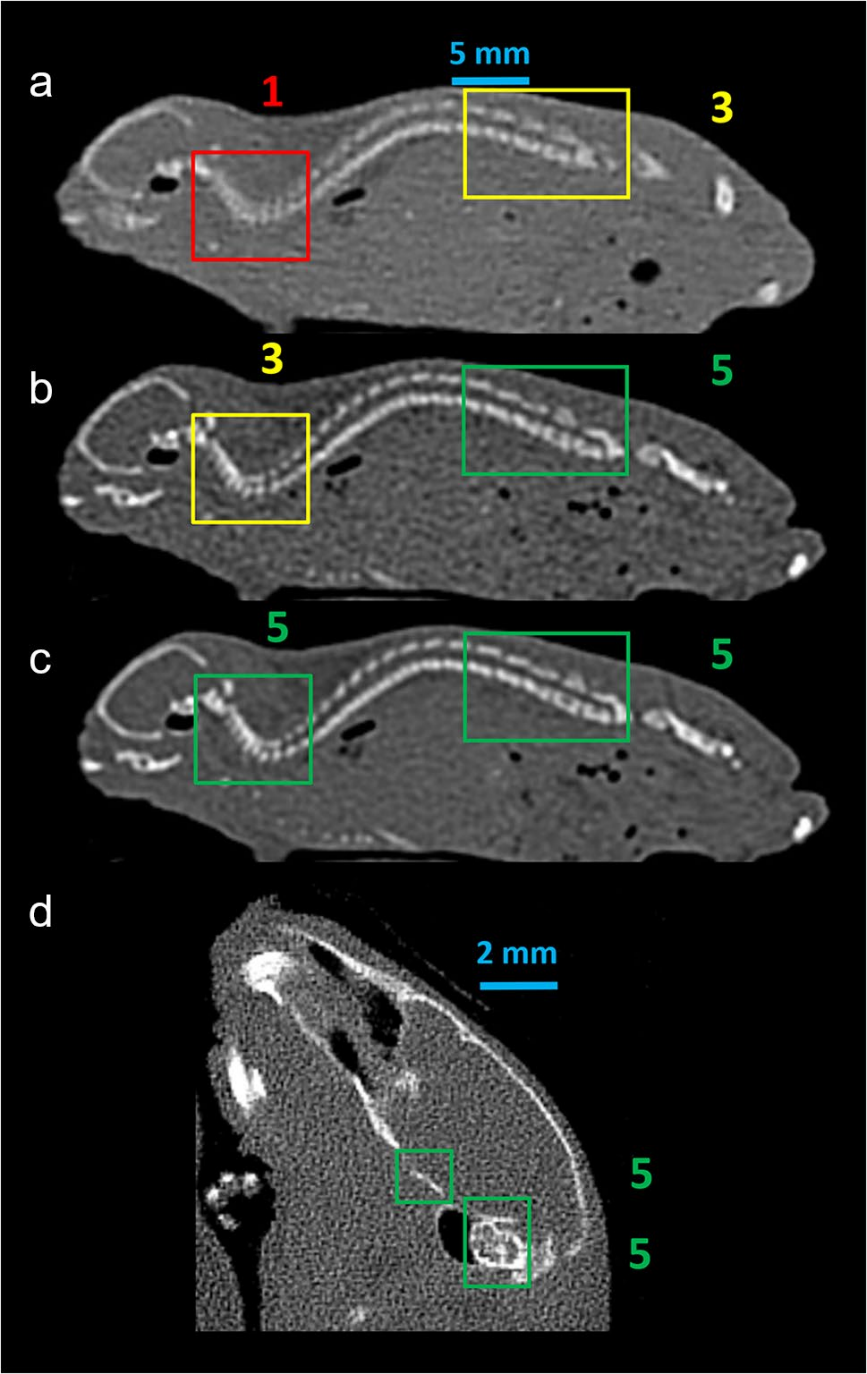
Semiquantitative analysis of bone detail visualization. Examples for semiquantitative analysis of bone detail visualization. 1 = no delineation, 2 = unsharp delineation, 3 = sharp delineation. **a**: EID-CT at CTDI_Vol_ = 5 mGy, **b**: PCD-CT at CTDI_Vol_ = 3 mGy, **c**: PCD-CT at CTDI_Vol_ = 10 mGy. CTDI_Vol_, volumetric CT dose index; EID-CT, energy-integrating detector CT; PCD-CT, photon-counting detector CT

Using the 0.2-mm reconstructions and the 0.6-mm coronal reformations of the cervical spine, two radiologists semiquantitatively evaluated four defined anatomic regions: (I) lumbar spine: spinal canal; (II) lumbar spine: transverse foramen; (III) cervical spine: transverse processes; and (IV) cervical spine: intervertebral discs. Radiologists assessed the visualization of details in consensus on a 3-point Likert scale (1 = no delineation, 2 = unsharp delineation, 3 = sharp delineation, Fig. 2). Readers were blinded towards the scanner and all dose settings and datasets were presented in random order.

### Evaluation of observer preference in SNR-matched pairs

To validate our results from SNR analyses, 20 SNR-matched doubles of EID-CT and PCD-CT series were generated and presented to two readers in random order with the task of selecting the reconstruction with better detail visualization.

Supplemental Fig. 1 shows a flow chart for our study including data acquisition, reconstruction, postprocessing, and data analysis.

### Statistical analysis

Continuous variables are reported as mean ± standard deviation and evaluated for normal distribution using Kolmogorov–Smirnov tests. Differences between both groups (EID-CT and PCD-CT) were analyzed by *t*-tests for independent samples if variables were normally distributed. Otherwise, non-parametric tests were used. To test the significance in the difference of proportions, the chi-squared test was applied. A *p* value < 0.05 was defined as significant. All analyses were performed on IBM Statistics SPSS 26.0.

## Results

### Image noise and signal-to-noise ratio

**Fig. 3:**
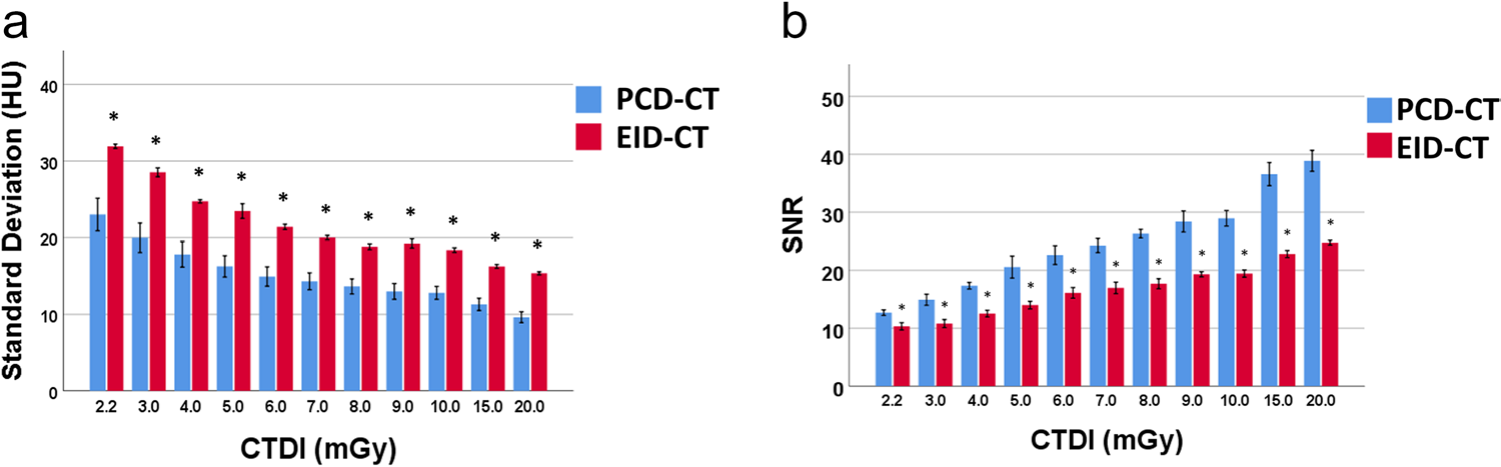
Comparison of image noise and SNR. (**a**) Image noise measured as standard deviation of CT values of air at various CTDI_Vol_ values. (**b**) Signal-to-noise ratio for various CTDI_Vol_ values.; *p value < 0.05. CTDI_Vol_, volumetric CT dose index; EID-CT, energy-integrating detector CT; HU, Hounsfield units; PCD-CT, photon-counting detector CT; SNR, signal-to-noise ratio

Image noise was significantly lower in PCD-CT reconstructions compared to EID-CT reconstructions at all CTDI_Vol_ values (all *p*’s < 0.05, Fig. 3A, Supplemental Table 1). Outperformance of PCD-CT consistently increased with CTDI_Vol_ and ranged from 27.9% (8.92/31.93; PCD-CT: 23.01 HU vs. EID-CT: 31.93 HU, absolute difference 8.92 HU) at a CTDI_Vol_ of 2.2 mGy to 37.3% (5.74/15.37; PCD-CT: 9.63 HU vs. EID-CT: 15.37 HU, absolute difference 5.74 HU) at a CTDI_Vol_ of 20 mGy.

Likewise, SNR was significantly higher in PCD-CT series compared to EID-CT at all CTDI_Vol_ values (all *p*’s < 0.01, Fig. 3b, Supplemental Table 2). Relative outperformance of PCD-CT series also consistently increased from 18.7% (2.37/12.7; PCD-CT: 12.7 vs. EID-CT: 10.3, absolute difference 2.37) at a CTDI_Vol_ of 2.2 mGy to 36.3% (14.11/38.87; PCD-CT: 38.87 vs. EID-CT: 24.76, absolute difference 14.11) at a CTDI_Vol_ of 20 mGy.

### Edge sharpness

**Fig. 4:**
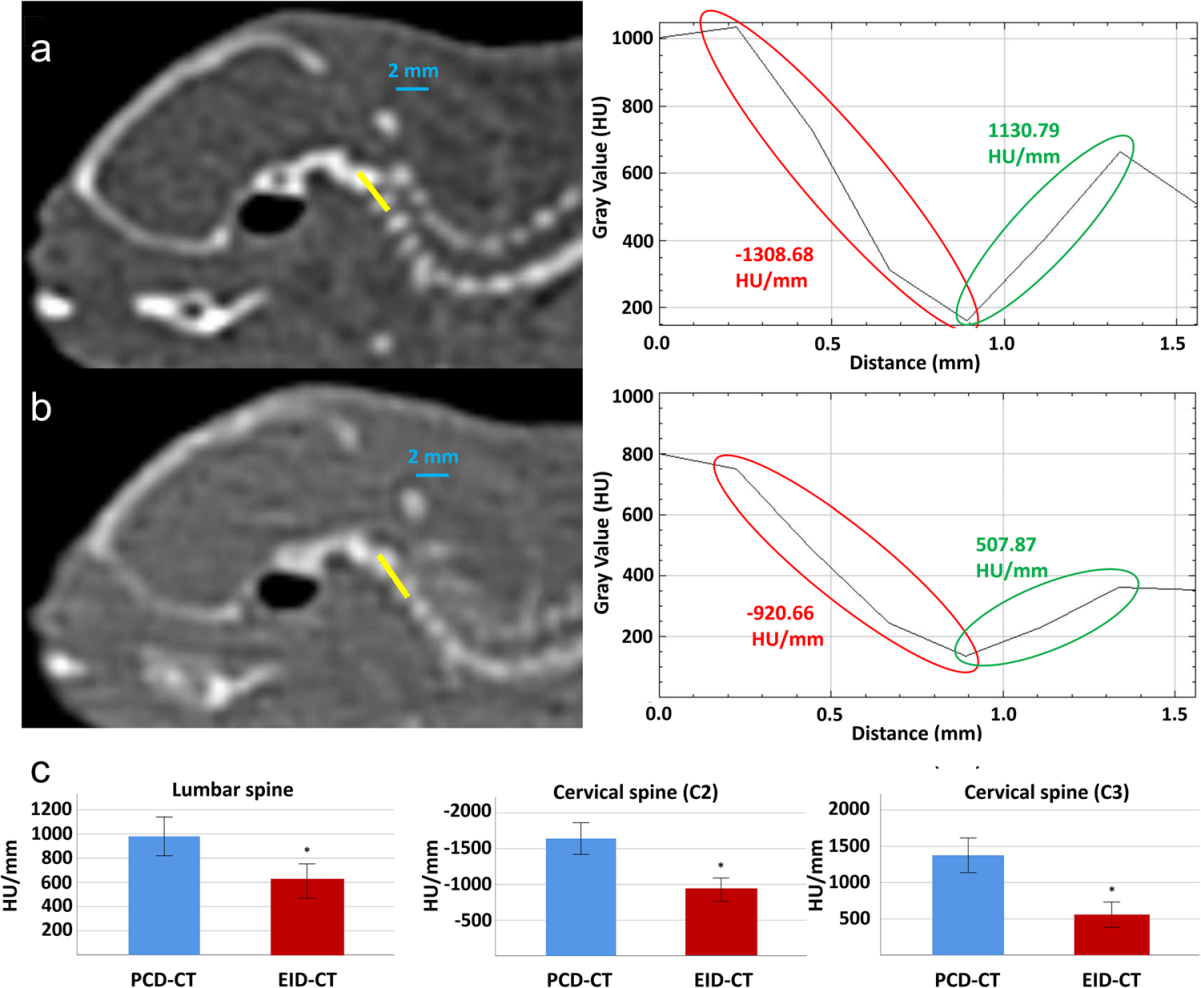
Comparison of edge sharpness. Examples of the quantification of edge sharpness. The first row (**a**) shows cervical spine at PCD-CT (CTDI_Vol_ = 10 mGy) with corresponding plots demonstrating slopes of CT values. Decrease and increase of CT values is highlighted in red/green. The second row (**b**) shows cervical spine at EID-CT (CTDI_Vol_ = 9 mGy) with corresponding plots. The third row (**c**) shows comparisons of edge sharpness between PCD-CT and EID-CT at three distinct anatomic locations (lumbar spine, cervical spine C2, cervical spine C3). CTDI_Vol_, volumetric CT dose index; EID-CT, energy-integrating detector CT; HU, Hounsfield units; PCD-CT, photon-counting detector CT

Quantitative analyses of edge sharpness revealed significant differences between EID-CT and PCD-CT with consistently steeper slopes for PCD-CT for all CTDI_Vol_ values (all *p*’s < 0.05, Fig. 4, Supplemental Table 3). At the lumbar spine, slopes had a range of 543–723 HU/mm for EID-CT and of 940–1042 HU/mm for PCD-CT. At the cervical spine, slopes had a range of 296–1170 HU/mm for EID-CT and 1126–1860 HU/mm for PCD-CT, respectively. As expected, there was no consistent relationship between CTDI_Vol_ values and measured slopes so average values across measurements at various CTDI_Vol_’s were used.

### Bone detail visualization

**Fig. 5:**
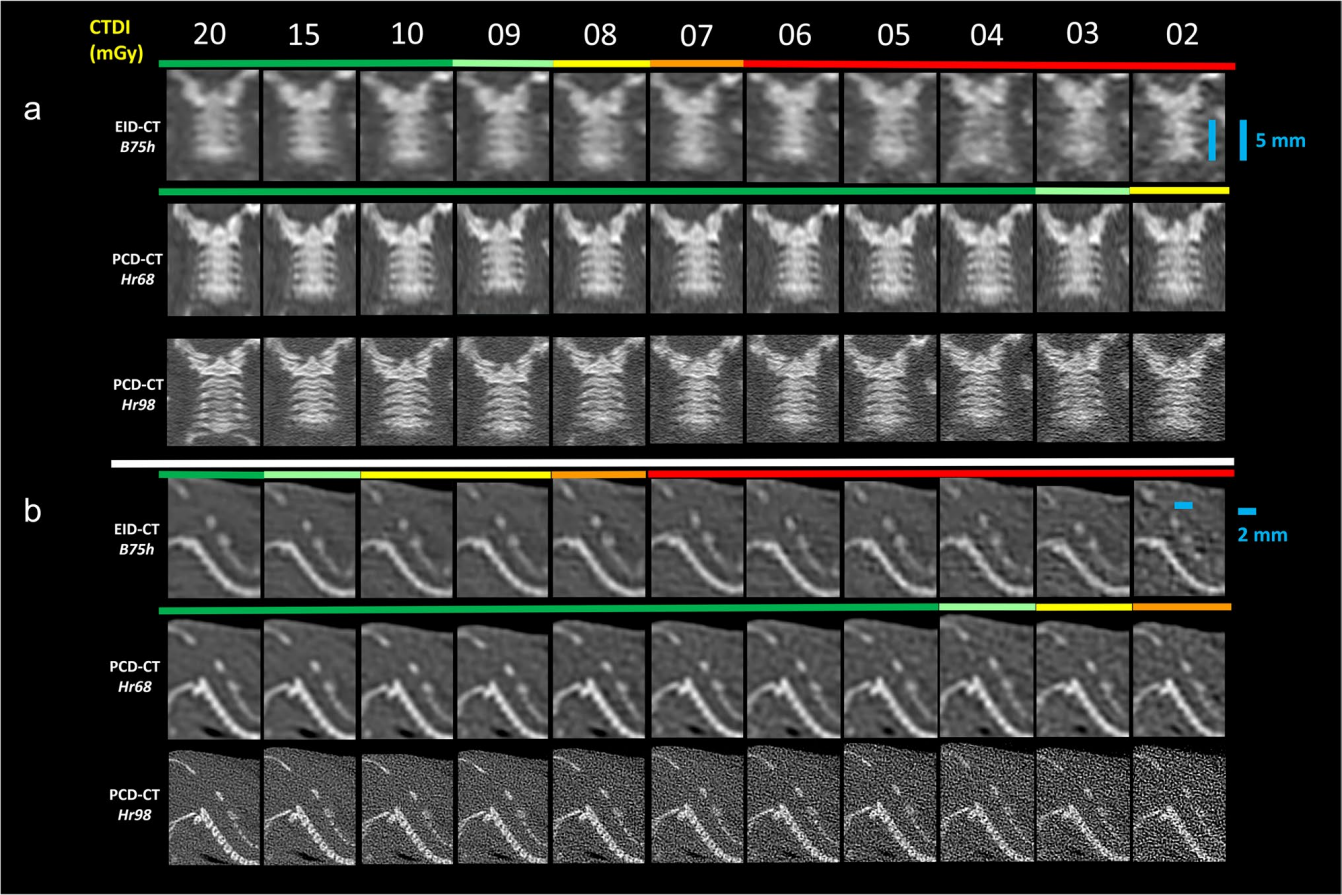
Semiquantitative evaluation of bone detail visualization on 0.6-mm coronal and 0.2-mm sagittal reformations of the cervical spine. (**a**) 0.6-mm coronal reformations of the cervical spine for the visualization of the transverse processes. (**b**) Sagittal 0.2-mm reconstructions of the cervical spine for visualization of the intervertebral discs. Colored lines indicate semiquantitative evaluation of two readers in consensus for delineation of transverse processes (**a**) and intervertebral discs (**b**) at various CTDI_Vol_ values; red, no delineation; yellow, unsharp delineation; green, sharp delineation. CTDI_Vol_, volumetric CT dose index; EID-CT, energy-integrating detector CT; PCD-CT, photon-counting detector CT; B75h, Hr68, and Hr98 are abbreviations for specific reconstruction algorithms

At the cervical spine, the clear visualization of intervertebral discs required a CTDI_Vol_ of 20 mGy and ≥ 4 mGy for EIC-CT and PCD-CT, respectively. Likewise, sharp delineation of transverse processes required ≥ 10 mGy and ≥ 4 mGy for EIC-CT and PCD-CT. At the lumbar spine, clear visualization of transverse foramina was observed at CTDI_Vol_ values ≥ 6 mGy and ≥ 3 mGy for EID-CT and PCD-CT, whereas sharp delineation of the lumbar spinal canal required CTDI_Vol_ values ≥ 4 mGy for EID-CT but was observed at all analyzed CTDI_Vol_ values for PCD-CT (cervical spine: Fig. 5; Video 1 lumbar spine: Supplemental Fig. 2).

Additional Hr98 reconstructions were performed for PCD-CT. These reconstructions are routinely available at PCD-CT single-pixel mode acquisitions and exhibit supreme spatial resolution and sharp delineation of tiny bone details. As these are not available for EID-CT, they were not used for comparison between scanners. Figure 5 and Video 1 demonstrate the cervical spine in coronal 6-mm reformations as examples of bone detail visualization for EID-CT and PCD-CT (Hr68 and Hr98) at different CTDI_Vol_ values.

### Observer preference in SNR-matched pairs

SNR-matching produced the following CTDI_Vol_-pairs for EID-CT and PCD-CT series, respectively: (20 mGy; 7 mGy) with SNR of 24.8 and 24.3; (15 mGy; 6 mGy) with SNR of 22.8 vs. 22.6; (10 mGy; 4 mGy) with SNR of 19.4 and 17.4; (6 mGy, 3 mGy) with SNR of 16.1 and 14.9 and (4 mGy; 2.2 mGy) with SNR of 12.5 and 12.7, for EID-CT and PCD-CT, respectively.

Evaluating SNR-matched pairs for visualization of bone details, reader 1 and reader 2 selected PCD-CT in 19/20 and 18/20 cases, respectively. Both ratios are significantly different from a 50% ratio (i.e., random choice, both *p*’s < 0.05).

## Discussion

In this study, we compared the visualization of finest bone details between a novel dual-source PCD-CT in single-pixel mode and EID-CT using a young mouse as a specimen. In dose-matched scans, PCD-CT series showed significantly lower image noise, higher SNR, and higher edge sharpness than EID-CT series. Using comparable linear reconstruction algorithms for both scanners, two radiologists considered the delineation of predefined bone structures (such as transverse foramen) as feasible at consistently lower CTDI_Vol_ values at PCD-CT than at EID-CT. In direct comparison of SNR-matched series, PCD-CT series were still considered superior in almost all cases.

Differences in image noise and SNR were observed across the analyzed CTDI_Vol_ spectrum and consistently in favor of PCD-CT and ranged from 27.9 to 37.3% and from 18.7 to 36.3% for image noise and SNR, respectively. These observations are consistent with the literature published to date [5, 6, 8, 9]. The higher differences in SNR of up to 60% reported by Grunz et al. [5] might be due to the slightly different definition of image noise in our study. While Grunz et al. used the standard deviation within subcutaneous fat to quantify image noise, we measured standard deviation within a large ROI in adjacent air. Due to the sparsity of subcutaneous fat in young mice, standard deviations within air were much more reproducible. Unlike some of the prior literature, our study solely relied on linear reconstruction algorithms to avoid any confounding by unaddressed differences in iterative algorithms, which might also have contributed to lower differences between PCD-CT and EID-CT in our study.

Our results confirm the dose-saving potential inherent in PCD-CT in single-pixel acquisition mode in comparison with EID-CT. Our results are in line with Grunz et al. [5] in that CTDI_Vol_ can be significantly lower in PCD-CT and still yield identical SNRs as EID-CT at higher CTDI_Vol_ values, but also extend those of prior studies: PCD-CT datasets with nominally comparable SNR (acquired at much lower CTDI_Vol_ values) are still considered superior by observers in the vast majority of cases. This suggests that dose-saving potential should considerably exceed that suggested by SNR difference.

Our study has several limitations. First, it is well known that standard deviations within ROIs incompletely reflect the degree and quality of image noise in a CT dataset and contain no information about its frequency distribution [10]. Nevertheless, this parameter is frequently chosen in image quality studies on clinical CT datasets and some excellent recent work has emphasized the close relationship between this simple parameter and more comprehensive measures of image noise for the types of image noise typically encountered in clinical CT scans [11, 12]. Second, the mice in our study were scanned without any other attenuating objects in the gantry—except for X-ray transparent casing and the patient table. Therefore, we cannot prove that our results will fully extend to scans of human anatomy with larger diameters or whole patient scans. Yet in those scenarios, scatter radiation will be considerably higher; due to the very low sensitivity of PCD-CT for scattered, low-energy photons, difference in image quality in favor of PCD-CT should even be accentuated. Third, we did not employ an UHR comb filter on the EID-CT with which pixel size can be “virtually” reduced thereby increasing spatial resolution. It is conceivable that such an increase in spatial resolution would have increased observer preference for EID-CT images; with the use of an UHR comb, however, dose efficiency of EID-CT would have been considerably worse and in all likelihood much more divergent CTDI_Vol_ values would have been compared as SNR equivalent. This would even strengthen our finding of the dose-saving potential of PCD-CT in single-pixel acquisition mode.

In conclusion, our results demonstrate the improved visualization of finest anatomic details using PCD-CT in comparison with EID-CT. This can be attributed to two overlapping effects, namely the better spatial resolution and the reduction of image noise at comparable CTDI_Vol_ values. Our results are further evidence that this improved visualization does not incur any dose penalty but can be unambiguously measured at comparatively lower radiation doses.

## Supplementary Information

Below is the link to the electronic supplementary material.

Supplementary file1 (RAR 23923 KB)

## Abbreviations

CTDI_Vol_: Volumetric computed tomography dose index
EID-CT: Energy-integrating detector CT
FoV: Field of view
HU: Hounsfield units
PCD-CT: Photon-counting detector CT
ROI: Region of interest
SNR: Signal-to-noise ratio
UHR: Ultra-high resolution

## Declarations

### Guarantor

The scientific guarantor of this publication is PD Dr. med. Florian Schwarz.

### Statistics and biometry

PD Dr. med. Florian Schwarz, PD Dr. med. Stefanie Bette, and Dr. Josua Decker have significant statistical expertise.

### Methodology

•prospective.

•experimental

•performed at one institution.

## References

[1] Flohr T, Petersilka M, Henning A, Ulzheimer S, Ferda J, Schmidt  B (2020). Photon-counting CT review. Phys Medica.

[2] Leng S, Bruesewitz M, Tao S (2019). Photon-counting detector CT: system design and clinical applications of an emerging technology. Radiographics.

[3] Zhou W, Lane JI, Carlson ML (2018). Comparison of a photon-counting-detector CT with an energy-integrating-detector CT for temporal bone imaging: a cadaveric study. AJNR Am J Neuroradiol.

[4] Leng S, Rajendran K, Gong H (2018). 150-μm spatial resolution using photon-counting detector computed tomography technology: technical performance and first patient images. Invest Radiol.

[5] Grunz J-P, Huflage H, Heidenreich JF (2021). Image quality assessment for clinical cadmium telluride-based photon-counting computed tomography detector in cadaveric wrist imaging. Invest Radiol.

[6] Gutjahr R, Halaweish AF, Yu Z (2016). Human imaging with photon counting-based computed tomography at clinical dose levels: contrast-to-noise ratio and cadaver studies. Invest Radiol.

[7] Schindelin J, Arganda-Carreras I, Frise E (2012). Fiji: an open-source platform for biological-image analysis. Nat Methods.

[8] Leng S, Yu Z, Halaweish A (2016). Dose-efficient ultrahigh-resolution scan mode using a photon counting detector computed tomography system. J Med Imaging (Bellingham).

[9] Symons R, Reich DS, Bagheri M (2018). Photon-counting computed tomography for vascular imaging of the head and neck: first in vivo human results. Invest Radiol.

[10] Boedeker KL, Cooper VN, McNitt-Gray MF (2007). Application of the noise power spectrum in modern diagnostic MDCT: Part I. Measurement of noise power spectra and noise equivalent quanta. Phys Med Biol.

[11] Christianson O, Winslow J, Frush DP, Samei E (2015). Automated technique to measure noise in clinical CT examinations. AJR Am J Roentgenol.

[12] Ahmad M, Jacobsen MC, Thomas MA, Chen HS, Layman RR, Jones AK (2020). A benchmark for automatic noise measurement in clinical computed tomography. Med Phys.

